# Comparable efficacy of bictegravir/emtricitabine/tenofovir alafenamide and doravirine/lamivudine/tenofovir disoproxil fumarate in therapy-naive Human Immunodeficiency Virus adults with very high baseline viraemia

**DOI:** 10.1099/jmm.0.002119

**Published:** 2026-04-10

**Authors:** Leonardo Calza, Maddalena Giglia, Claudio Rigamonti, Alberto Zuppiroli, Domenico Marzolla, Isabella Bon, Pierluigi Viale

**Affiliations:** 1 Department of Medical and Surgical Sciences, Unit of Infectious Diseases, S.Orsola Hospital, IRCCS Azienda Ospedaliero-Universitaria di Bologna, Bologna, Italy; 2 Unit of Microbiology, S.Orsola Hospital, IRCCS Azienda Ospedaliero-Universitaria di Bologna, Bologna, Italy

**Keywords:** bictegravir, doravirine, effectiveness, high viraemia, tolerability

## Abstract

**Introduction.** Clinical trials and observational studies of co-formulated regimens bictegravir/emtricitabine/tenofovir alafenamide (BIC/F/TAF) and doravirine/lamivudine, tenofovir disoproxil fumarate (DOR/3TC/TDF) demonstrated potent efficacy in both antiretroviral therapy-naive and -experienced patients, but data on efficacy in naive people with high-level Human Immunodeficiency Virus (HIV) viraemia are still lacking.

**Hypothesis.** BIC/F/TAF and DOR/3TC/TDF have comparable effectiveness in naive people with high-level viraemia.

**Aim.** To compare the effectiveness of BIC/F/TAF and DOR/3TC/TDF in naive people with high-level viraemia.

**Methodology.** We performed a retrospective cohort study of adult people living with HIV, who were naive to antiretroviral therapy, had baseline HIV-1 RNA >500,000 copies ml^−1^ and initiated the co-formulated regimen BIC/F/TAF or DOR/3TC/TDF. Virological efficacy, changes in immunological parameters and safety profiles after 12 months of treatment were evaluated.

**Results.** Inclusion criteria were met by 78 patients: 43 in the BIC/F/TAF group and 35 in the DOR/3TC/TDF group. Baseline characteristics were similar in both groups: median age was 45.2 years, median HIV RNA was 5.95 log_10_, median CD4 T lymphocyte count was 383 cells mm^−3^ and 22 patients (28%) had HIV RNA >10^6^ copies ml^−1^. After 12 months, virological efficacy was comparable: HIV RNA <20 copies ml^−1^ was obtained in 40 patients (93%) in the BIC/F/TAF group and in 31 (89 %) in the DOR/3TC/TDF group. The median increase in CD4 T lymphocyte count was comparable between groups (+139 and +117 cells mm^−3^, respectively), such as incidence of adverse events, while median increase in low-density lipoprotein cholesterol and weight gain was significantly greater in the BIC/F/TAF group (weight change, +1.64 kg vs. +0.85 kg; *P*=0.013).

**Conclusion.** In this real-life cohort, BIC/F/TAF and DOR/3TC/TDF showed high and comparable virological efficacy in naive patients with baseline HIV RNA above 500,000 copies ml^−1^.

## Introduction

It is not completely clear whether a very high baseline Human Immunodeficiency Virus (HIV) viral load (above 500,000 copies ml^−1^) can impair the virological response to combination antiretroviral therapy (cART), and clinical data about the virological efficacy of currently recommended initial regimens in people living with HIV (PLWH) with very high pre-therapy viraemia are still limited.

Triple co-formulated combinations bictegravir/emtricitabine/tenofovir alafenamide (BIC/F/TAF) and doravirine/lamivudine/tenofovir disoproxil fumarate (DOR/3TC/TDF) are two antiretroviral regimens currently recommended in antiretroviral therapy-naive PLWH by European AIDS Clinical Society Guidelines [1].

In a double-blind, phase-3, randomized 1489 study, at week 96, BIC/F/TAF achieved virological suppression in 88 % of previously untreated adults and was non-inferior to co-formulated combination dolutegravir, abacavir and lamivudine, with no treatment-emergent resistance and a better gastrointestinal tolerability [2,3]. In a phase-3, randomized 1490 study, at week 96, virological suppression with BIC/F/TAF was achieved in 84 % of untreated individuals and was non-inferior to the triple regimen dolutegravir, emtricitabine and tenofovir alafenamide, in association with a good and comparable safety profile [4,5]. Week 144 results from 1489 and 1490 studies confirmed non-inferior efficacy of BIC/F/TAF compared to both dolutegravir-containing regimens in naive PLWH, and this regimen was well tolerated with additional exposure [6].

However, in 1489 and 1490 trials, median baseline HIV viral load in enrolled patients assigned to BIC/F/TAF was about 4.4 log_10_ copies ml^−1^, and only 17 % of these subjects had pre-therapy HIV RNA >100,000 copies ml^−1^ [2,4].

The non-nucleoside reverse transcriptase inhibitor doravirine, in association with lamivudine and tenofovir disoproxil fumarate at week 96, demonstrated non-inferior efficacy and superior profiles for neuropsychiatric safety and lipid values in treatment-naive adults in comparison with efavirenz plus emtricitabine and tenofovir disoproxil fumarate in the DRIVE-AHEAD phase-3, randomized study [7,8].

In the DRIVE-FORWARD phase-3 randomized trial, at week 96, doravirine showed a superior virological efficacy and a more favourable lipid profile compared to ritonavir-boosted darunavir, both associated with tenofovir disoproxil fumarate plus emtricitabine or abacavir plus lamivudine, in therapy-naive individuals [9,10]. High virological efficacy and favourable safety profiles for doravirine at week 96 were maintained through to week 192 in PLWH who continued or switched to doravirine-containing regimen [11], but in DRIVE-AHEAD and DRIVE-FORWARD studies too, median baseline HIV RNA was 4.4 log_10_ copies ml^−1^, and patients with HIV RNA >100,000 copies ml^−1^ were only about 20 % of the study population [7,9].

So, data from clinical trials make evident the shortage of information about the virological efficacy of these antiretroviral regimens in previously untreated PLWH with very high pre-therapy viraemia.

The aim of our observational, exploratory study is to evaluate the efficacy of BIC/F/TAF and DOR/3TC/TDF in antiretroviral therapy-naive PLWH with baseline HIV viral load above 500,000 copies ml^−1^.

## Methods

We performed a single-centre, observational, retrospective study of adult PLWH naive to antiretroviral therapy, referred to our Clinic of Infectious Diseases from January 2020 through December 2023, with baseline plasma HIV RNA >500,000 copies ml^−1^, and who initiated the daily single-tablet regimen BIC/F/TAF or DOR/3TC/TDF. The choice of the initial regimen was clinician-based, and there were no default criteria for assigning one treatment over the other. Candidates were those who received at least one dose of BIC/F/TAF or DOR/3TC/TDF. All enrolled subjects were followed for 12 months.

All enrolled subjects were tested for primary viral resistance mutations by genotypic test. Exclusion criteria were having received a previous antiretroviral treatment for >7 days, genotypic testing showing reduced sensitivity to bictegravir and/or doravirine and/or emtricitabine and/or lamivudine and/or tenofovir, pregnancy, alcohol abuse or intravenous drug use, alanine aminotransferase (ALT) or aspartate aminotransferase >80 U l^−1^, creatinine >1.5 mg dL^−1^ and treatment with medications or herbal supplements known to affect the pharmacokinetics of current antiretroviral drugs. Alcohol abuse and intravenous drug dependence were defined as a daily alcohol consumption of >30 g and >1 intravenous drug use within 6 months before starting the dual regimen, respectively.

Demographic, clinical and laboratory data were recorded at the start of therapy and at 6-month intervals during the 12-month follow-up. All the available data were collected during routine clinical care.

HIV-1 quantification by real-time PCR was performed using the Abbott Alinity-m test (Abbott Molecular Inc, Des Plaines, IL, USA), which uses a dual target/dual probe design against highly conserved target regions of the HIV-1 genome (integrase and LTR regions). The dynamic range of quantification of the Alinity m HIV-1 assay is 20 to 107 copies ml^−1^.

All samples for haematological and biochemical tests were processed by the Central Laboratory of the S. Orsola hospital.

The primary endpoint was the virological success at month 12. Results were evaluated both in intention-to-treat (ITT) and per-protocol (PP) analyses. The ITT analysis included all subjects with at least one dose of drug taken and participants who were lost to follow-up, discontinued or changed antiretroviral therapy, died or had no data at month 12 were considered failures. The PP analysis included all the ITT patients except those with loss of follow-up, who had no 12-month data or discontinued or changed antiretroviral regimen for reasons other than a virological failure. The secondary endpoints included changes in CD4+lymphocyte count, CD4+/CD8+ lymphocyte ratio, metabolic parameters, weight and body mass index (BMI) at month 12.

Virological success was defined as plasma HIV RNA <20 copies ml^−1^ at month 12 and virological failure as a confirmed plasma HIV RNA >20 copies ml^−1^ at month 12. Genotypic testing was performed in case of virological failure, using targeted next-generation sequencing and Illumina NovaSeq platform (Novogene, Cambridge, UK), and Stanford University HIV Drug Resistance algorithm was employed to interpret the resistance mutations. In genotypic tests, the following regions were sequenced: reverse transcriptase, protease and integrase. Regarding safety evaluations, the occurrence of adverse events (AEs) and treatment discontinuation for adverse events were reported by the clinicians in medical records and gathered from their review. The Division of AIDS (DAIDS) Table for Grading the Severity of Adult and Pediatric Adverse Events, version 2.1, was employed for the evaluation of AEs. The DAIDS grading table provides an AE severity grading scale ranging from grades 1 to 5. Grade 1 indicates a mild event; grade 2 indicates a moderate event; grade 3 indicates a severe event; grade 4 indicates a potentially life-threatening event; grade 5 indicates death.

The adherence to the current therapy was carefully checked on the outpatient visits by self-reported questionnaires. The adherence questionnaires employed in the study were clinical reports showing the number of drug doses omitted in the last 30 days.

## Statistical analysis

Data are presented as median with interquartile range (IQR) and mean with sd for descriptive data and continuous variables, while comparisons between two categorical variables with more than two modalities were performed by Fisher’s exact test. The significance of changes in all the considered variables was assessed using the paired Student’s t-test. Depending on the distribution, the t-test or the Wilcoxon rank-sum test was used to analyse continuous data. D’Agostino’s *K*^2^ test was employed as a normality test to gauge the compatibility of given data with the null hypothesis that the data are a realization of independent, identically distributed Gaussian random variables. Cumulative risks of 12-month virological and immunological endpoints were estimated using Kaplan–Meier analysis. All statistical tests were bilateral, and a *P* value of<0.05 was considered statistically significant. Excel 2007–2013 (Microsoft, Redmond, WA, USA) was used to input the data, and SPSS 23.0 (IBM, Armonk, NY, USA) was employed for statistical analysis.

## Results

Study inclusion criteria were met by 78 patients who were enrolled in the study. Median age (IQR) was 45.2 (26, 71) years, 66 patients (84.6%) were males and 69 (88.5 %) were Caucasian. The median baseline log_10_ HIV RNA (IQR) was 5.95 (5.71, 6.12), and 22 patients (28.2%) had HIV RNA >10^6^ copies ml^−1^. Median baseline CD4+ lymphocyte count was 383 cells mm^−3^, 20 subjects (25.6%) had a CD4+ lymphocyte count <200 cells mm^−3^ and 15 (19.2 %) had acquired immune deficiency syndrome (AIDS) diagnosis.

At baseline, 17 patients (21.8%) had one or more comorbidities, and the most common comorbidities were hypertension (11.5 % of patients), neuropsychiatric disorders (7.7%), osteoporosis (6.4%) and diabetes mellitus (2.3%). Chronic hepatitis B was diagnosed in one case (2.3%). Comedications were reported in 20 cases (25.6%), and the most common concomitant drugs were proton pump inhibitors (15.4%), benzodiazepines (14.1%) and ACE inhibitors (9%).

Baseline characteristics of study patients exhibited a general homogeneity across both groups and are summarized in Table 1.

**Table 1.** Baseline characteristics of the enrolled patients

	BIC/F/TAF	DOR/3TC/TDF	*P*
No. of patients	43	35	
Males, no. (%)	36 (83.7)	30 (85.7)	0.447
White subjects, no. (%)	38 (88.4)	31 (88.6)	0.709
Age (years), median (IQR), mean (sd)	46.4 (26, 65) 45.7 (13.8)	43.2 (24, 59) 42.1 (11.7)	0.067 [t]
HIV transmission risk category, no. (%):			
IDU	3 (7)	1 (2.9)	0.551
MSM	29 (67.4)	25 (71.4)	0.178
Heterosexual	11 (25.6)	9 (25.7)	0.368
Log_10_ HIV RNA (copies mL), median (IQR), mean (SD)	5.97 (5.71, 6.12) 5.88 (1.22)	5.93 (5.66, 6.07) 5.76 (1.43)	0.228 [W]
Patients with HIV RNA >1,000,000 copies ml^−1^, no. (%)	13 (30.2)	9 (25.7)	0.098
CD4+ lymphocyte count (cells mm^−3^), median (IQR), mean (sd)	371 (48, 791) 384 (122)	402 (92, 668) 413 (145)	0.509 [W]
CD4+/CD8+ lymphocyte ratio, median (IQR), mean (sd)	0.43 (0.09, 0.72) 0.46 (0.19)	0.48 (0.12, 0.87) 0.51 (0.22)	0.582 [W]
AIDS diagnosis, no. (%)	9 (20.9)	6 (17.1)	0.117
Patients with CD4+ lymphocyte count <200 cells mm^−3^, no (%)	11 (25.6)	9 (25.7)	0.293
Patients with >1 comorbidities, no (%)	10 (23.2)	7 (20)	0.188
Patients with hypertension, no (%)	5 (11.6)	4 (11.4)	0.679
Patients with diabetes mellitus, no. (%)	1 (2.3)	0	0.999
Patients with chronic hepatitis B, no. (%)	1(2.3)	0	0.999
Patients with chronic hepatitis C, no. (%)	0	0	
Weight (kg), median (IQR), mean (sd)	73.6 (57.2, 91.3) 74.2 (12.5)	74.9 (60.2, 88.7) 76.4 (13.1)	0.385 [t]
BMI (kg m^−2^), median (IQR), mean (sd)	24.6 (20.4, 28.3) 24.8 (0.6)	25.2 (21.3, 30.1) 25.7 (0.7)	0.491 [t]
Total cholesterol (mg dL^−1^), median (IQR), mean (sd)	183 (165, 219) 188 (55)	188 (167, 225) 192 (61)	0.629 [t]
LDL cholesterol (mg dL^−1^), median (IQR), mean (sd)	127 (95, 164) 132 (21)	132 (99, 171) 139 (28)	0.446 [t]
HDL cholesterol (mg dL^−1^), median (IQR), mean (sd)	48 (33, 67) 44 (13)	50 (36, 73) 48 (15)	0.273 [t]
Triglycerides (mg dL^−1^), median (IQR), mean (sd)	191 (135, 266) 186 (79)	187 (126, 249) 178 (65)	0.184 [t]
Creatinine (mg dL^−1^), median (IQR), mean (sd)	0.83 (0.64, 1.09) 0.85 (0.22)	0.71 (0.56, 0.94) 0.69 (0.21)	0.129 [t]

[t], Student’s t-test; [W], Wilcoxon rank-sum test.

HDL, high-density lipoprotein; IDU, injection drug users; LDL, low-density lipoprotein; MSM, men who have sex with men.

Notably, we found that the median time from HIV-1 infection confirmation to cART initiation was less than 14 days in all patients.

Following the initiation of antiretroviral therapy, by month 6, the proportion of patients with virological success in the BIC/F/TAF group was 88.4 % (38/43) by the ITT analysis and 90.5 % (38/42) by the PP analysis. Virological efficacy was comparable to 85.7 % (30/35) by the ITT analysis and to 88.2 % (30/34) by the PP analysis in the DOR/3TC/TDF group (*P*=0.102 and 0.218, respectively).

After 12 months, the proportion of patients with virological success in the BIC/F/TAF group was 93 % (40/43) by the ITT analysis and 97.6 % (40/41) by the PP analysis. Virological efficacy was comparable to 88.6 % (31/35) by the ITT analysis and to 93.9 % (31/33) by the PP analysis in the DOR/3TC/TDF group (*P*=0.101 and 0.257, respectively) (Table 2). Kaplan–Meier estimates of the 12-month risk of the virological endpoints (treatment failures and having HIV RNA <20 copies ml^−1^ at month 12) by treatment strategy, and risk difference with 95% confidence interval (95 % CI), are outlined in Table 3.

**Table 2.** Virological and immunological results at month 12 (descriptive analysis)

	BIC/F/TAF	DOR/3TC/TDF	*P*
No. of enrolled patients	43	35	
Treatment failures, no. (%):			
Discontinuations due to AE	2 (4.7)	2 (5.7)	0.386
Virological failures	1 (2.3)	2 (5.7)	0.301
Missing Data	0	0	
Virological successes (patients with HIV RNA <20 copies ml^−1^):			
ITT analysis (%)	40/43 (93)	31/35 (88.6)	0.101
PP analysis (%)	40/41 (97.6)	31/33 (93.9)	0.257
Median (IQR) and mean (sd) change from baseline to month 12 in CD4+ lymphocyte count (cells mm^−3^)	+139 (+62, +256) +135 (+97)	+117 (+41, +228) +122 (+91)	0.301 [t]
Median (IQR) and mean (sd) change from baseline to month 12 in CD4+/CD8+ lymphocyte ratio	+0.21 (+0.11, +0.42) +0.23 (+0.09)	+0.24 (+0.09, + 0.47) +0.25 (+1.1)	0.268 [t]

[t], Student’s t-test; [W], Wilcoxon rank-sum test.

**Table 3.** Virological and immunological results at month 12 (Kaplan–Meier analysis)

	BIC/F/TAF	DOR/3TC/TDF	Risk difference
No. of enrolled patients	43	35	
Cumulative risk of treatment failures, (%) (95 % CI)			
Discontinuations due to AE	4.7 (3.1, 6.2)	5.7 (4.2, 7.1)	-1 (-2.1, -0.1)
Virological failures	2.3 (1.2, 3.5)	5.7 (3.9, 7.2)	-3.4 (-5.6, -1.5)
missing data	0	0	
Cumulative risk of HIV RNA <20 copies ml^−1^, (%) (95 % CI)			
ITT analysis	93 (89.1, 96.3)	88.6 (83.2, 92.1)	4.4 (1.8, 6.9)
PP analysis	97.6 (92.9, 99.1)	93.9 (90.7, 96.3)	3.7 (2.2, 5.8)
Cumulative risk of >100 CD4+ lymphocyte mm^−3^ increase from baseline to month 12, (%) (95 % CI)	96.2 (93.9, 99.9)	95.8 (93.2, 98.6)	0.4 (0.21, 0.57)
Cumulative risk of >0.2 increase in CD4+/CD8+ lymphocyte ratio from baseline to month 12, (%) (95 % CI)	85.2 (81.4, 89.5)	83.8 (80.9, 86.5)	1.4 (0.6, 2.7)

Overall, adherence to antiretroviral therapy was >95 % in 38 out of 43 (88.4 %) patients in the BIC/F/TAF group and in 30 out of 35 (85.7 %) patients in the DOR/3TC/TDF group (*P*=0.493).

After 12 months, three treatment failures (7%) were observed in the BIC/F/TAF group: one discontinuation (2.3%) due to virological failure and two discontinuations (4.7%) due to non-serious adverse events (Table 2).

In the unique case of virological failure, HIV RNA was 690 copies ml^−1^ at month 12, and genotypic analysis demonstrated no resistance mutations. This patient switched antiretroviral therapy to tenofovir alafenamide/emtricitabine/darunavir/cobicistat and reached a plasma HIV RNA <20 copies ml^−1^ within 3 months. In this subject, adherence to antiretroviral treatment at month 12 was lower than 95 %.

There were two discontinuations for non-serious adverse events: headache in one case (2.3%) and sleeping disturbances in one case (2.3%).

After 12 months, four treatment failures (11.4%) were observed in the DOR/3TC/TDF group: two discontinuations (5.7%) due to virological failure and two discontinuations (5.7%) due to non-serious adverse events (Table 2).

In the first case of virological failure, HIV RNA was 1350 copies ml^−1^ at month 12, and genotypic analysis demonstrated no resistance mutations. This patient switched antiretroviral therapy to BIC/F/TAF and reached a plasma HIV RNA <20 copies ml^−1^ within 3 months. In the second case of virological failure, HIV RNA was 880 copies ml^−1^ at month 12, and genotypic analysis demonstrated no resistance mutations. This patient switched antiretroviral therapy to tenofovir alafenamide/emtricitabine plus dolutegravir and reached a plasma HIV RNA <20 copies ml^−1^ within 3 months. In both subjects, adherence to antiretroviral treatment at month 12 was lower than 95 %.

There were two discontinuations for non-serious adverse events: headache in one case (2.8%) and gastrointestinal symptoms in one case (2.8%).

After 12 months, a significant and comparable increase in CD4+ cell count was observed in both groups. The median increase (IQR) in CD4+ lymphocyte count was +139 cells mm^−3^ (+62, +256) in the BIC/F/TAF group and +117 cells mm^−3^ (+41, +228) in the DOR/3TC/TDF group (*P*=0.301). A similar change was also noted in CD4/CD8 ratios: the median increase (IQR) from baseline to month 12 in CD4+/CD8+ lymphocyte ratio was +0.21 (+0.11, +0.42) in the BIC/F/TAF group and +0.24 (+0.09, +0.47) in the DOR/3TC/TDF group (*P*=0.268) (Table 2). Kaplan–Meier estimates of the 12-month risk of the immunological endpoint (>100 CD4+ lymphocyte mm^−3^ increase and >0.2 increase in CD4+/CD8+ lymphocyte ratio from baseline to month 12) by treatment strategy, and risk difference with 95 % CI, are outlined in Table 3.

During the 12-month follow-up, a significantly greater elevation in median concentrations (IQR) of total cholesterol and low-density lipoprotein (LDL) cholesterol was reported in the BIC/F/TAF group than in the DOR/3TC/TDF group. The median change in total cholesterol was +42 (+18, +68) mg dL^−1^ in the former group and +9 (−12, +27) mg dL^−1^ in the latter group (*P*<0.001). The median change in LDL cholesterol was +21 (+6, +43) mg dL^−1^ in the former group and −4 (−21, +19) mg dL^−1^ in the latter group (*P*<0.001).

Instead, there was no significant difference in median variations of high-density lipoprotein (HDL) cholesterol, triglycerides and glucose between the two groups at month 12. The median change in HDL cholesterol was +5 (−6, +11) mg dL^−1^ in the BIC/F/TAF group and −3 (−8, +7) mg dL^−1^ in the DOR/3TC/TDF group (*P*=0.519). The median change in triglycerides was +44 (+26, +65) mg dL^−1^ in the former group and +35 (+17, +58) mg dL^−1^ in the latter group (*P*=0.217). The median change in glucose was +0.11 (+0.03, +0.18) mg dL^−1^ in the former group and +0.09 (+0.02, +0.14) mg dL^−1^ in the latter group (*P*=0.178).

In terms of kidney and liver function tests, there was no significant difference in median change of ALT and creatinine between the two groups at month 12. The median change in ALT was +9 (−2, +14) U l^−1^ in the BIC/F/TAF group and +2 (−10, +11) U l^−1^ in the DOR/3TC/TDF group (*P*=0.244). The median change in creatinine was +0.08 (+0.01, +0.12) mg dL^−1^ in the former group and +0.13 (+0.06, +0.19) mg dL^−1^ in the latter group (*P*=0.448).

A significantly higher increase in median value (IQR) of body weight was observed at month 12 in the BIC/F/TAF group compared to the DOR/3TC/TDF group: +1.64 (+0.96, +3.12) kg vs. +0.85 (+0.21, +1.54) kg, respectively (*P*=0.013). As a consequence, a significantly higher increase in median value (IQR) of BMI was observed at month 12 in the BIC/F/TAF group compared to the DOR/3TC/TDF group: +0.75 (+0.31, +1.04) kg m^−2^ vs. +0.33 (+0.12, +0.76) kg m^−2^, respectively (*P*=0.027).

No serious adverse events were reported during the 12-month follow-up, and no grade 3 or 4 clinical events or laboratory abnormalities were recorded in both groups. Overall, the incidence of adverse events was comparable in the BIC/F/TAF group (11/43, 25.6%) and in the DOR/3TC/TDF group (9/35, 25.7%) (*P*=0.772). The most common adverse events in the former group were headache (5 patients; 11.6%), sleeping disturbances (4 patients; 9.3%) and diarrhoea with/without abdominal pain (2; 4.6%). The most common adverse events in the latter group were headache (4 patients; 11.4%) and diarrhoea with/without abdominal pain (3; 8.6%). All reported adverse events were grade 1 or 2.

## Discussion

The baseline level of HIV viral load has been considered traditionally one determining factor associated with the success of first-line antiretroviral treatment, similar to various other parameters, including baseline CD4+ cell count, pre-existing drug resistance, coinfections and adherence. Effectively, in multivariate analyses of cohort studies, the pre-therapy level of HIV RNA is an independent predictor of full HIV RNA suppression [12,14], although inconsistent results have been obtained in randomized clinical trials [15,20].

Patients with baseline HIV RNA >100,000 copies ml^−1^ usually take longer to achieve full viral suppression, and this slower decrease in plasma viral load might increase the chance of treatment-emergent drug resistance on certain antiretroviral drugs and favour a more prolonged viral transmission. In addition, high baseline HIV RNA may be a marker of advanced HIV disease such as low CD4+ cell count, and this condition is usually associated with a shorter life expectancy [21,22].

The rate of decline in HIV RNA during initial antiretroviral therapy may differ significantly between treatment classes, but the clinical significance of these variations is still unknown, and clinical data about virological efficacy of different antiretroviral agents in PLWH with very high baseline HIV RNA are lacking [23,24].

A meta-analysis of 21 randomized trials showed a significantly lower percentage of patients with HIV RNA <50 copies ml^−1^ at week 48 when the baseline HIV RNA level was >100,000 copies ml^−1^, and this difference in efficacy was consistent across trials of different nucleoside reverse transcriptase (NRTI) backbones and third agents, represented by a non-nucleoside reverse transcriptase inhibitor (NNRTI), a protease inhibitor (PI) or an integrase strand transfer inhibitor (INSTI) [25].

Clinical studies that assessed virological efficacy of different antiretroviral regimens in naive individuals with high baseline HIV viral load are limited and often lead to contrasting results.

A retrospective cohort study, including 758 treatment-naive subjects in China starting an NNRTI-based regimen from 2009 to 2013, showed that very high baseline viraemia (over 500,000 copies ml^−1^) was associated with delayed virological suppression, incomplete viral suppression and viral rebound [26]. On the contrary, in a multicentre, retrospective study including 220 naive patients in the USA with baseline HIV RNA >100,000 copies ml^−1^ and starting cART between 2005 and 2016, NNRTI recipients were more likely to achieve virological suppression by 6 months compared to those initiating cART containing PIs or INSTIs [27].

In a Spanish nationwide cohort of 4,186 naive individuals starting cART between 2015 and 2018, baseline HIV RNA >100,000 copies ml^−1^ was significantly associated with a higher risk of low-level viraemia and virological failure even in participants receiving INSTIs [28].

In the RESPOND Cohort Study, including 4,310 treatment-naive participants who initiated a triple regimen in 2014–2020, baseline HIV RNA >100,000 copies ml^−1^ and CD4+ T cell count <200 mm^−3^ were negatively associated with virological suppression at week 48 and 96, with significantly higher rates of viral blips, low-level viraemia and residual viraemia. These results were consistent in those starting integrase inhibitors versus other regimens [29].

A retrospective, multicentre study evaluated 162 individuals with advanced HIV infection initiating cART with dolutegravir/lamivudine/abacavir (DOL/3TC/ABC) or BIC/F/TAF in 2019–2020. At baseline, median HIV viral load was 5.22 log_10_ copies ml^−1^, and 27 % of patients had HIV viral load >500,000 copies ml^−1^. At week 48, virological suppression rate was comparable: 73.5 % in DOL/3TC/ABC group and 85.7 % in BIC/F/TAF group (*P*=0.178), but regimen discontinuation was less prevalent among patients receiving BIC/F/TAF [30].

The CoRIS cohort study assessed the effectiveness of BIC/F/TAF compared to other first-line antiretroviral regimens in 314 naive individuals with advanced HIV infection initiating cART in 2019–2020. At baseline, median HIV viral load ranged between 250,000 and 273,000 copies ml^−1^, 62–69 % of patients had HIV RNA over 100,000 copies ml^−1^, and 32–42 % had an AIDS diagnosis. In multivariable analysis, people with AIDS-defining conditions initiating cART with BIC/F/TAF achieved higher rates of virological suppression after 24 weeks than other regimens [31].

In the Rainbow prospective pilot study enrolling 30 individuals with advanced HIV infection and starting BIC/F/TAF within 7 days from HIV diagnosis, median baseline HIV RNA was 6 log_10_ copies ml^−1^. After 48 weeks, the proportion of participants with HIV RNA <50 copies ml^−1^ was 27/30 (90 %), and no discontinuations due to virological failure or toxicity were observed [32].

The BIC-NOW phase IV, single-arm, clinical trial enrolled 208 naive patients who initiated cART with BIC/F/TAF on the day of their first visit to the HIV specialist between 2020 and 2022. At baseline, 22.6 % of subjects had AIDS, the median HIV RNA was 5.6 log_10_ copies ml^−1^ and 43.3 % had HIV RNA >100,000 copies ml^−1^. The efficacy at week 48 was 84.1 % by ITT analysis and 98.3 % by PP analysis, and the regimen was discontinued in only two subjects [33].

In our study, initial cART with triple regimen BIC/F/TAF or DOR/3TC/TDF showed comparable efficacy in a cohort of 78 antiretroviral therapy-naive people with baseline very high viraemia (HIV RNA >500,000 copies ml^−1^), with a high rate of virological suppression after 12 months (93% and 89 % in the ITT analysis, respectively). Only three patients had confirmed virological failure, but genotype testing showed no resistance-associated mutations for INSTIs, NNRTIs or NRTIs.

These regimens also demonstrated a favourable and comparable immunologic response at month 12 (+139 and + 117 CD4+ cells mm^−3^, respectively), in association with a low and comparable incidence of adverse events. No significant changes in liver and kidney function tests were reported in both groups, while the median increase in LDL cholesterol and weight gain was significantly greater in the BIC/F/TAF group.

Several limitations are present in our study, so the results must be interpreted with caution: first, the retrospective observational design, with some confounding factors which could potentially influence the choice of treatment, even though the baseline characteristics of patients were comparable across all treatment groups; second, the limited sample size, the short duration of the observation period and the data extraction from the medical records which can be occasionally incomplete or inaccurate; third, our study is underpowered for non-inferiority, and there is a chance of not finding a difference in viral suppression efficacy even if there is one, so this is an exploratory study; and finally, some aspects, such as the control of viral replication in the central nervous system or other reservoirs and the effect on the immune activation markers or peripheral fat, were not evaluated in the present study.

To conclude, in clinical practice, the single-tablet regimens BIC/F/TAF and DOR/3TC/TDF represent an effective and safe initial regimen in people with HIV with baseline very high-level viraemia (over 500,000 copies ml^−1^), with a comparable virological and immunological effectiveness. However, larger randomized and observational studies are needed to better evaluate the efficacy and tolerability of these regimens in naive individuals with an initial high HIV viral load.

## Abbreviations

AEs: adverse events
AIDS: acquired immune deficiency syndrome
ALT: alanine aminotransferase
BIC/F/TAF: bictegravir/emtricitabine/tenofovir alafenamide
BMI: body mass index
cART: combination antiretroviral therapy
95% CI: 95% confidence interval
DAIDS: Division of AIDS
DOR/3TC/TDF: doravirine/lamivudine/tenofovir disoproxil fumarate
HDL: high-density lipoprotein
HIV: human immunodeficiency virus
INSTI: integrase strand transfer inhibitor
IQR: interquartile range
ITT: intention-to-treat
LDL: low-density lipoprotein
NNRTI: non-nucleoside reverse transcriptase inhibitor
NRTI: nucleoside reverse transcriptase
PI: protease inhibitor
PLWH: people living with HIV
PP: per-protocol
